# Relating Paramagnetic Properties to Molecular Parameters of Humic Acids Isolated from Permafrost Peatlands in the European Arctic

**DOI:** 10.3390/molecules29010104

**Published:** 2023-12-23

**Authors:** Evgeny Lodygin, Roman Vasilevich, Evgeny Abakumov

**Affiliations:** 1 Institute of Biology, Komi Science Center, Ural Branch, Russian Academy of Sciences, 28, Kommunisticheskaya St., 167982 Syktyvkar, Russia; vasilevich.r.s@ib.komisc.ru; 2 Department of Applied Ecology, Faculty of Biology, St. Petersburg State University, 16th Liniya V.O., 29, 199178 St. Petersburg, Russia; e_abakumov@mail.ru

**Keywords:** soil organic matter, free radicals, EPR spectroscopy, Histosols

## Abstract

Free radicals (FRs) are intermediate participants in the transformation process of soil organic matter, and free radical activity is a fundamental property of humic substances. The aim of this work was to conduct a comparative study of the paramagnetic properties of humic acids (HAs) isolated from Histosols by electron paramagnetic resonance (EPR) spectroscopy. The studied Histosols are found in permafrost peatlands in four natural geographic subzones of the European Arctic (from forest tundra to northern tundra). The results obtained showed that in anaerobic conditions on the peatlands in the tundra zone, the formation of semiquinone-type radicals occurs through the reduction of quinone fragments of HAs and leads to an increase in the concentration of paramagnetic centres within HAs. PCA analysis allowed us to reveal relationships between the properties of the initial raw peat samples, the molecular composition of the isolated HAs, and their paramagnetic parameters. It was found that FR localization occurs predominantly on aromatic fragments of lignin nature, which are confined to the low molecular weight fraction of HAs. The g-factor values of the EPR spectra of HAs indicate the presence of carbon- and oxygen-centred FRs in the HA structure, with a predominance of the latter.

## 1. Introduction

Many studies on soil organic matter confirm the important role of semiquinone-type organic free radicals (FRs) in biochemical processes [1,2]. FRs are atoms or molecular groupings with an unpaired electron, which gives them paramagnetic properties and allows them to be detected by magnetic resonance spectroscopy. In inorganic systems, SRs are generally short-lived, but in complex organic compounds, they become stable, especially in the presence of aromatic nuclei. Semiquinone radicals are a classic example of such radicals. Modern concepts indicate that these radicals are intermediates in the formation of humic substances in soils [3,4], and free-radical activity is a fundamental property of soil organic matter (SOM) [5,6].

The use of electron paramagnetic resonance (EPR) to study organic matter in soils provides a variety of information about the structure of radical-containing substances [7,8]. The paramagnetic properties of humic acids (HAs) depend on zonal bioclimatic factors and the level of anthropogenic impact [9,10,11]. Previous studies have shown a positive correlation between the degree of humification and FR concentration [5]. In addition, the content of paramagnetic centres in HA is determined by the degree of condensation and the colour coefficient [12]. As a result of experiments, it was assumed that the formation of HAs occurs during the condensation of polyphenols, amino acid residues, etc., carried out through the stage of free-radical structures. The EPR method has high sensitivity and allows for obtaining original information on the structure of radical-containing substances. The study of the free-radical structure of biopolymers, including humic substances, makes it possible to estimate their ability to undergo polymerisation reactions [13].

Arctic wetlands contain significant carbon stocks stored in SOM [14]. Permafrost degradation in this region leads to the release of melted SOM and increased carbon cycling [15]. This is also facilitated by geomorphological processes such as thermokarst and erosion, which expose deep layers of organic matter [16]. In addition, these areas are traditional reindeer grazing areas, which affects the stability and biodegradability of SOM as reindeer trample lichens and mosses, which are the main drivers of plant communities in the Arctic zone [17].

Studies using modern physicochemical methods to analyse the molecular structure of HAs in mineral soils have now been published [5,6,9]. However, studies of HAs in European Arctic peatlands are rare and lack systematicity. Peatlands are specific natural soil-like organic formations where the nature of humic compounds has been least studied [3]. Analysis of study results showed that both natural factors and agricultural use of soils have a significant influence on the concentration of HAs in humic compounds [1,10,18,19]. In this context, the aim of our work was to investigate the paramagnetic properties of HA samples isolated from peat hummocky bogs in four natural geographic subzones of the European Arctic and to reveal the relationship between paramagnetic properties and molecular parameters of HAs.

## 2. Results and Discussion

### 2.1. The EPR Data of HAs

**Figure 1:**
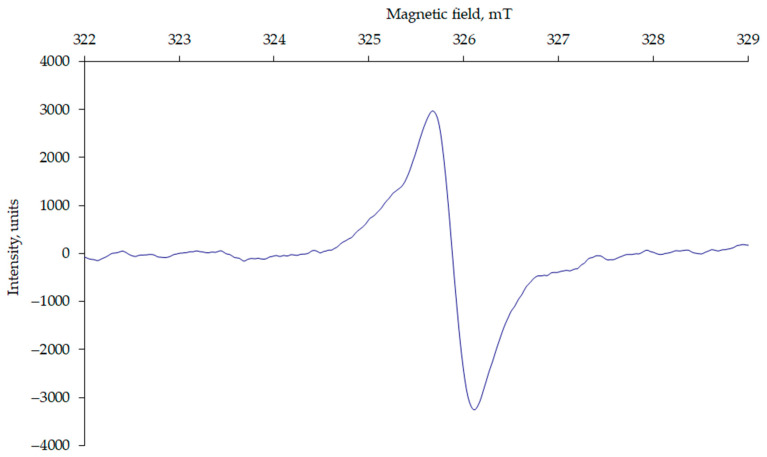
Typical EPR spectrum, exemplified by HAs from Hemic Folic Cryic Histosol (P-1, hor. Hi).

The EPR spectra of the HA samples (Figure 1) showed a broad line with a g-factor close to that of a free electron (g = 2.0023), indicating the presence of a strongly delocalized molecular orbital in the HA structure. The g-factor of the EPR spectra is a useful parameter for identifying the nature of FRs. Radicals with g-factors in the range of 2.0020 to 2.0050 are organic radicals [20]. Studies have shown that g-factors less than 2.0030 are carbon-centred radicals, whereas the g-factor of oxygen-centred radicals is greater than 2.0040 [21].

The EPR signal has the shape of a Lorentzian curve, which transitions to a Gaussian distribution on the wings. This indicates that, in addition to spin and spin-lattice interactions, the unpaired electron has sufficient freedom for spin-spin contacts [22,23]. A characteristic feature of HAs is the redistribution of electron density in their molecular π-orbitals, which accounts for the paramagnetic properties of HAs [3,24].

Calculations of the integral intensity of the absorption line allowed us to estimate the concentrations of unpaired electrons in the samples (Table 1). The results showed that the concentration of paramagnetic centres in HA preparations is higher than in raw peat samples [6], due to the presence of a large number of aromatic and other polyconjugated structures in HA molecules, which may contain unpaired electrons.

The CPC in the investigated HA samples usually has a bimodal character, with maxima on the surface and permafrost horizons (Table 1). This is due to solar irradiation of the upper layer and a high degree of peat decomposition in the permafrost horizons, resulting in a higher content of aromatic and other polyconjugated structures in the peat HA composition, on which unpaired electrons can delocalize. Below 60 cm, there is an increase in the paramagnetic activity of HAs caused by the change in the botanical composition of the original peat and an increase in the proportion of wood residues and sedges enriched with syringyl- and guaiacyl-propane units of lignin fragments, which contribute to the stabilization of FRs and the formation of paramagnetic properties of HAs.

The g-factor values of the EPR spectra of the HAs from the peatlands studied vary between 2.00350 and 2.00372, indicating a mixture of carbon- and oxygen-centred radicals in the HA structure, with the latter predominating. The general trend of g-factor values observed for peatland HAs (Table 1) is not as pronounced, probably due to the different nature of the sediments. There is only a tendency for g-factor values to decrease with depth. The decrease in index values is explained by the delocalization of the unpaired electron from O,N-substituted structures (mainly semiquinones surrounded by O-containing fragments) to gradually increasing condensed aromatic fragments. Such a phenomenon has been observed in the natural carbonization processes of humic substances from compost, peats, and various types of coal [18,25,26]. Experimental data for living peat-forming plants show relatively high values of g-factor: 2.0043 for *Carex* sp. and 2.0044 for *Sphagnum* sp. [27]. Such high g-factor values have not been observed under the regenerative conditions of Arctic peatlands, even for the upper peat layers.

### 2.2. Principal Component Analysis

The paramagnetic properties of peat HAs are determined by a number of factors. Therefore, PCA was performed to determine the influence of the molecular composition of HAs isolated from peat samples with different chemical properties on the free radical concentration and g-factor of HAs.

The PCA results explained 63.21% of the total variability of the HA properties. The dimensionality of the 19 input variables was reduced by PCA to two principal components with eigenvalues greater than two: the first axis (PC1) explained 46.34% and the second axis (PC2) explained 16.87% of the total variability (Table 2).

**Figure 2:**
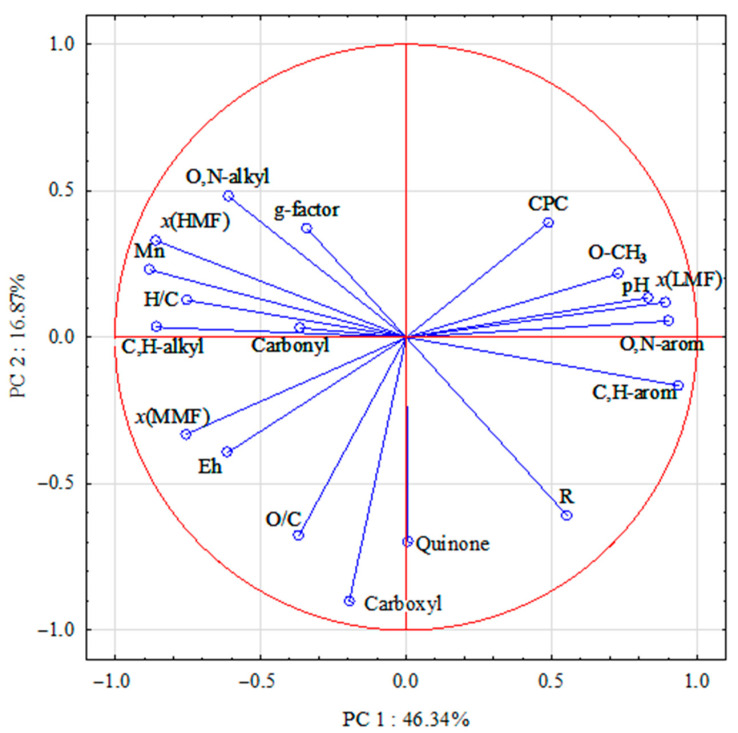
Projection of HA parameters using PCA: abbreviations as in Table 1 and Table 2.

The results indicate that the paramagnetic properties of HAs depend on both the molecular composition of the HAs themselves and the properties of the original raw peat samples (Figure 2).

The PCA results show that PC1 is positively related to CPC, low molecular weight fraction (*x*(LMF)), C atom content of methoxyl (O-CH_3_) and aromatic fragments (O,N-arom and C,H-arom), degree of peat decomposition (*R*), acidity (pH) and negatively related to g-factor, medium (*x*(MMF)) and high molecular weight (*x*(HMF)) fractions, acidity (pH) and is negatively related to g-factor, medium (*x*(MMF)) and high molecular weight (*x*(HMF)) fractions, number average molecular weight (*Mn*), content of alkyl fragments (C,H-alkyl and O,N-alkyl), H/C element ratios and peat redox potential (Eh).

The proximity of the CPC, the content of C atoms of methoxyl and aromatic fragments in the HA structure, and the *x*(LMF) of HAs in the graph may indicate the localization of unpaired electrons predominantly on “lignin” fragments in the HA composition. This is also confirmed by the significant correlation between the concentration of paramagnetic centres and the content of O-CH_3_ groups (*r* = 0.40, *n* = 43, *r*_cr_ = 0.30), O,N-arom (*r* = 0.32, *n* = 43, *r*_cr_ = 0.30) and C,H-arom fragments (*r* = 0.33) as well as the *x*(LMF) (*r* = 0.46, *n* = 43, *r*_cr_ = 0.30). The findings are consistent with results indicating that the HMF of HAs consists mainly of C atoms of aliphatic structures, whereas the LMF of HA tends to contain aromatic fragments [28]. Studies by Shi Y. et al. [29] also showed that a high proportion of oxygen-centred FRs is characteristic of low molecular weight HA samples (<3.5, <7, and <14 kDa).

A negative relationship between CPC and Eh values of native peat is observed, due to a shift in the equilibrium towards the formation of semiquinone radicals from quinone fragments as the redox potential decreases:

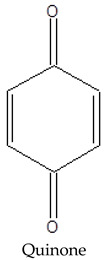
$\overset{\;+\bar{e}\;}{\to }$ $\overset{\;-\bar{e}\;}{\leftarrow }$
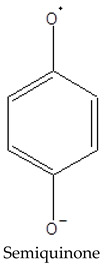


The peat layers of Arctic bogs are predominantly moderately and weakly reducing due to high humidity and the presence of anaerobic processes. Under these conditions, the humification of plant litter decreases, which increases the FR content, since in such regimes, paramagnetic centres of the semiquinone type are formed by the reduction of quinones. At the same time, semiquinone in the form of radical ions is most stable at higher pH values [5,30]. This may be one of the reasons for the positive relationship between CPC and pH in peat samples (Figure 2).

The close relationship between the g-factor and the content of O,N-substituted alkyl fragments of HA is confirmed by a significant correlation coefficient between these parameters (*r* = 0.45, *n* = 43, *r*_cr_ = 0.30). A statistically significant inverse relationship was found between the g-factor and the C-atom content of aromatic fragments. These results are in agreement with those obtained for HAs from the Baltic-type raised bog [31]. The effect of a decrease in the g-factor of semiquinone radicals observed for HAs with increasing aromaticity [32] was confirmed and theoretically explained on the basis of quantum mechanical calculations of the g-factor for model semiquinone radicals [33].

On the second PC2 axis, a significant negative relationship was observed for the degree of peat decomposition, O/C ratio, and the content of carboxyl groups, and quinone fragments (Figure 2). This is due to the fact that an increase in the degree of decomposition of peat-forming plants, which are GC precursors, is accompanied by an increase in the proportion of O-containing structures.

## 3. Materials and Methods

### 3.1. Study Area

**Figure 3:**
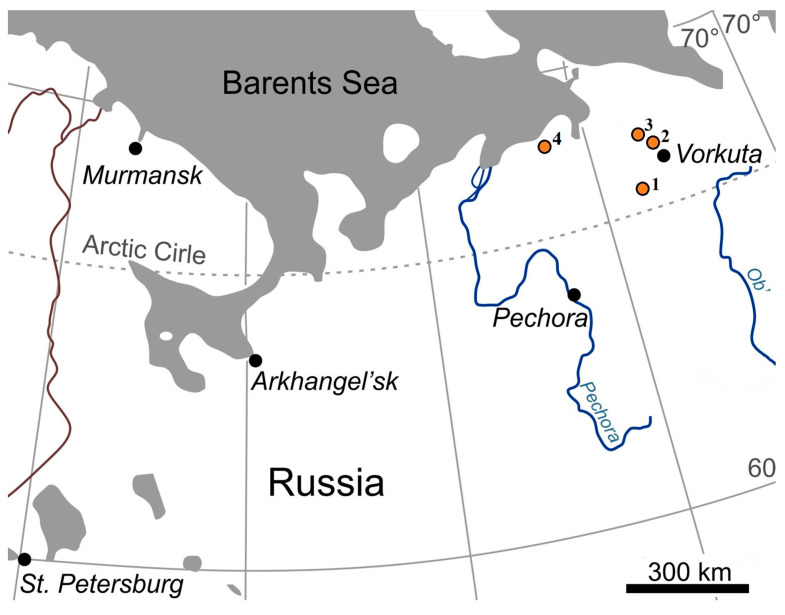
The study area: 1—forest tundra; 2—southern tundra; 3—transitional ecotone northern tundra and southern tundra; 4—northern tundra.

Four key wetland ecosystem polygons in the Arctic Belt were selected for this study (Figure 3), located in the Vorkuta District of the Komi Republic (KR) and the Nenets Autonomous Okrug (NAO).

These polygons represent key zonal biomes and are located in different areas of the Bolshezemelskaya tundra, including the forest tundra (Plot-1, Usa River basin, KR), the southern tundra (Plot-2, Khasyrei-Ty-Vis River basin, KR, and NAO border), the transitional ecotone of the northern and southern tundra (Plot-3, watershed of the Padimei-Ty-Vis and Korotaikha rivers, NAO), and the northern tundra (Plot-4, eastern part of the Pechora Bay of the Barents Sea, NAO). The studies were conducted in an area with massive islands and continuous permafrost [34].

According to climatic zoning, the study area belongs to the eastern subarctic subzone [35]. The climate here is dominated by Arctic air masses, with continentality increasing inland. The annual air temperature amplitude reaches 31–35 °C. The average annual air temperature in this area varies from minus 4 to minus 9 °C. The climatic conditions in this area are characterized by harsh conditions. Winter lasts about 250 days, and the frost period (when the average daily temperature is below minus 20 °C) lasts 80–95 days. The snow cover lasts from 230 to 240 days. The period of positive temperatures lasts from 100 to 115 days.

The moisture regime in the area is characterized by a high precipitation rate, moderate evaporation, and high air and soil moisture. Annual rainfall decreases from south to north and ranges from 500 to 360 mm. Maximum rainfall occurs during the warm season, usually in August, and accounts for about 70 percent of the total annual rainfall (200 to 300 mm). Depending on the type of vegetation cover, the depth of the roof of perennially frozen rocks in loamy soils varies and can range from 0.7–0.8 to 2.0–3.0 m or more [36]. Moss-shrub and coarse-shrub (bird and willow) tundras are widespread on plateaus. In areas with poor drainage, sedges and flat-bumped bogs are found. In the Arctic, vegetation cover is affected by the recurring seasonal processes of thawing and freezing of soil water, fluidity of over-watered clay soils, soil frost heaving, crack formation, snow corrasion of the soil surface, turf breaks, and spotting [37,38]. Reindeer grazing and caterpillar transport have a major impact on vegetation cover [17].

### 3.2. Sampling Sites

#### 3.2.1. Forest Tundra

In the forest tundra, the studies were conducted within the flat-bumpy-mossy complex on Hemic Folic Cryic Histosol—Plot-1. In the following, the names of the soils are given in brackets according to the international WRB classification [39].

Plot-1: 67°03′ N, 62°56′ E, the plot is located 7 km southwest of the Seida railway station on a hill, 10–12 m in diameter and 0.5–1.0 m high. The micro-relief is slightly hilly. The vegetation cover is dominated by *Polytrichum commune*, *Pleurozium schreberi*, *Dicranum elongatum*, *Flavocetraria nivalis*, *Cladonia* (*C. arbuscula*, *C. rangiferina*, *C. coccifera*, *C. gracilis*, and *C. crispata*), *Empetrum hermaphroditum*, *Vaccinium vitis-idaea*, *Rubus chamaemorus*, *Vaccinium uliginosum*, and *Betula nana* along the edges of the hill.

The bog massif under consideration occupies a high terrace above the floodplain in the left-bank part of the Sedyakha River valley (a right-bank tributary of the Usa River). The absolute height of the terrace is about 90 m a.s.l., and the height difference at the watershed of the Sedyakha and Seyda Rivers is 100–160 m a.s.l. The bog massif is a boggy marsh massif, which is a boggy marshland. The peat bog is a ridge-hollow complex with thermokarst lakes. Peat hummocks of various shapes prevail within the bog boundaries; the height of hummocks is from 1 to 3 m. The studied bog is characterized by a well-defined microrelief, largely caused by permafrost processes. Approximately 60% of the massif is occupied by rises or hills, the rest being occupied by hollows, sinks, watered runoff hollows, and secondary lakes. The hills are rounded or oval in shape and their cross-sectional dimensions vary from 10–15 to 20–45 m. The surface is flat or finely punctuated. As a result of permafrost degradation, saucer-shaped depressions with hygrophyte vegetation are formed on some hillocks. The thickness of the seasonally thawed layer varies from 40 to 50 cm. The peat is dark brown throughout its thickness, with a high and medium degree of decomposition, and is of the humic type.

Data on the composition and physico-chemical properties of peat soils and HA preparations isolated from them are presented in Table 3. A more detailed characterization and description of the molecular properties of HAs have been published earlier [38,40].

#### 3.2.2. South Tundra

The study area is located in the southern tundra on the eastern macro slope of the Tarju and Vorkuta River watersheds, with a few streams and small rivers belonging to the Vorkuta River basin.

Plot-2: Fibric Folic Cryic Histosol, 67°45′ N, 63°18′ E. The plot is located on a flat peat plateau in the NAO, 28 km NW of Vorgashor, 3.5 km west of Lake Lek’yamboty, in the basin of the Lek’yambo-Ty-Vis River. The surface of the plateau has a tuberous microrelief; *Ledum decumbens*, *Rubus chamaemorus* dominate the vegetation cover, and the moss cover consists of *Polytrichum commune*, *Pleurosium schreberi*, species g. *Dicranum,* and g. *Cladonia*.

The study area is located in the Bolshezemelskaya tundra in the north-eastern part of the Pechora Lowland. Low hills and ridges, apparently of glacial and glacio-marine origin, dominate most of the area, alternating with wide gullies and lake basins. The hydrogeophysical network in the study area is rather dense, mainly due to small lakes. These are mainly small water bodies with a small surface area. The lakes are mainly located in the inter-block joints and therefore have pronounced depressions and mineral-free shores.

#### 3.2.3. Ecoton North Tundra—South Tundra

Studies in the ecotone zone of the northern tundra and the southern tundra were carried out on the soil of the Hemic Folic Cryic Histosol—Plot-3.

Plot-3: 68°02′ N, 62°43′ E; the plot is located on a flat hillock at the edge of a flat bog with a lake complex. The hill has a complex in shape, 15–20 m wide, 20–25 m long, and 0.7–1.5 m high. Microprofile of the hill: diameter 30–80 cm, height 10–15 cm. Interhilly spaces are irregularly shaped, up to 30–50 cm in diameter. The shrub layer consists of *Ledum decumbens*, *Betula nana*, *Empetrum nigrum*, *Vaccinium vitis-idaea,* and *Rubus chamaemorus*. Shrubs mainly occupy the surface of the hillocks and the peripheral part of the peat hillock, including its sloping surface. The interhilly spaces are mainly occupied by moss-lichen vegetation with dicranial and polytrichous mosses and various lichen species. The depth of the upper limit of the permafrost is 33–44 cm, depending on the micro-relief.

#### 3.2.4. North Tundra

In the northern tundra, studies were carried out on the Barents Sea coast within the flat-topped peat mound–hollow complex: Fibric Folic Cryic Histosol—Plot-4.

Plot-4: 68°35′ N, 55°55′ E; the plot is located in the centre of a ridge of the flat-bumpy bog. The shape of the hill is complex, polygonal, elongated from north to south, with sharp depressions of hollow and lake depressions. The hill has frost cracks crossing the ridges and bare areas without vegetation cover, d = 0.3–0.7 m. The bog is characterised by a well-defined micro-relief, largely caused by permafrost processes. The relief of the hills is shallow- to medium-hilly. The diameter of the hills varies from 50 to 100 cm. The shape of the hills varies from elongated oval to polygonal, bounded on the sides by lacustrine-hollow depressions. The diameter of the ridge at the sampling site is about 8 m, with a height of 0.5–0.7 m. Vegetation is represented by *Ledum decumbens*, *Rubus chamaemorus*, *Andromeda*, *Empetrum nigrum*, and *Vaccinium vitis-idaea*, as well as lichens and bryophyte mosses. The depth of the permafrost is 29–30 cm.

### 3.3. Methods

The method recommended by the International Humic Substances Society [41] was used for the extraction of HAs. HAs were extracted from 50 g of dry peat samples by double extraction with a 0.1 M NaOH solution at a ratio of 1:10 for complete extraction of HAs. The mixture was centrifuged for 1 h at 10,000 rpm on a SIGMA 2-16 KL ultracentrifuge (Sigma Laborzentrifugen GmbH, Osterode am Harz, Germany). The HA was then precipitated with a 6 M HCl solution to pH 1.0. HA was further purified from ash constituents using a mixture of 0.1 M HCl and 0.3 M HF. The ash content of HA preparations does not exceed 1%. To remove low molecular weight compounds, the HAs were dialyzed, then dried at 35 °C in a laboratory oven with forced convection, ground, and sieved through a 0.1 mm sieve.

The elemental composition of HAs was determined on a CHNSO analyser EA 1110 (Carlo-Erba, Cornaredo, Italy) at the Chromatography Common Use Centre (Institute of Biology, Syktyvkar, Russia) according to the certified methods of quantitative chemical analysis NN 88-17641-004-RA.RU.310657-2016 and 88-17641-116-01.00076-2011.

Registration of EPR spectra of HA samples was carried out at the Research Centre “Diagnostics of structure and properties of nanomaterials”—Kuban State University on the spectrometer JES FA 300 (JEOL, Tokyo, Japan) in the X-band. Measurement conditions: microwave power 1 mW, amplitude of high-frequency modulation 0.06 mTl. Manganese (II) oxide, with a known radical content, was used as an external standard to determine the concentration of paramagnetic centres (CPC) and as a reference for calculating the g-factors of the investigated HA samples. CPC in HA samples was determined by comparing the relative signal intensities of the sample and the standard using JES-FA swESR software v. 3.0.0.1 (JEOL, Tokyo, Japan). The absolute error in the determination of CPC by the EPR method is up to 10% [9].

Bivariate correlation analyses were performed using Pearson’s product-moment correlation coefficient (*r*), and statistical significance was assessed using the Neyman-Pearson (normal distribution) approach. The technically observed value of the coefficient (based on n pairs) was compared with the critical value (*r*_cr_) for a two-tailed test and a significance level of 0.05.

Principal component analysis (PCA) was performed to determine correlations between paramagnetic and molecular parameters of HAs using Statistica v. 12.1 (Dell, Round Rock, TX, USA). The number of factors extracted from the variables was determined by Kaiser’s rule. The first two principal components with an eigenvalue greater than two were retained for this criterion. All statistical analyses were performed at the specified significance level of *p* ≤ 0.05.

## 4. Conclusions

In the present work, the paramagnetic properties of HA preparations isolated from Histosols of four polygons of Arctic Belt peat ecosystems were investigated. These polygons are key zonal biomes and are located in different areas of the Bolshezemelskaya tundra, including forest tundra, southern tundra, northern and southern tundra ecotone, and northern tundra.

The results showed that the redox conditions of peatlands as well as the molecular parameters of HAs determine their paramagnetic properties. The peat layers of Arctic peat bogs have predominantly moderate and weak redox conditions due to high humidity and the occurrence of anaerobic processes. Under these conditions, FRs of the semiquinoid type are formed by the reduction of quinone fragments of HAs, which leads to an increase in the content of paramagnetic centres in the composition of HAs. At the same time, their concentration in HAs usually has a bimodal character of distribution along the profile of the studied peatlands, with maxima on the surface and permafrost horizons.

PCA of the obtained data set showed that the localization of unpaired electrons occurs predominantly on “lignin” fragments, which are restricted to the low molecular weight fraction of peat HAs. The g-factor values of the EPR spectra of HAs indicate the presence of carbon- and oxygen-centred radicals in the HA structure, with a predominance of the latter.

## Figures and Tables

**Table 1:** Paramagnetic properties of HAs from Arctic Histosols.

Horizon	Depth, cm	CPC ^1^, ×10^15^ spin/g	g-Factor ^2^
Forest–tundra subzone			
Hemic Folic Cryic Histosol (P-1)			
Hi	0–10	3.76	2.00354
He1	10–20	2.41	2.00352
He2	20–40	1.88	2.00350
Hef1	40–60	1.32	2.00353
Hef2	60–80	1.34	2.00352
Hef3	80–100	5.79	2.00356
Hef4	100–150	6.03	2.00354
Hef5	150–175	1.97	2.00350
Chgf	175–200	1.18	2.00351
South tundra			
Fibric Folic Cryic Histosol (P-2)			
O	0–10	4.21	2.00360
Hi1	10–15	1.93	2.00356
Hi2	15–20	5.30	2.00366
Hi3	20–26	1.05	2.00363
Hif1	26–40	2.55	2.00360
Hif2	40–50	4.33	2.00366
Hif3	50–60	4.33	2.00362
Hef1	80–100	4.16	2.00358
Hef2	120–140	5.82	2.00356
Hef3	160–180	4.84	2.00363
Hif4	200–220	3.20	2.00360
Hif5	240–260	6.22	2.00362
Hif6	260–280	4.62	2.00359
Ecoton north tundra—south tundra			
Hemic Folic Cryic Histosol (P-3)			
Hi	0–10	4.05	2.00360
He	10–20	2.45	2.00365
Hi1	20–30	3.47	2.00363
Hi2	30–33	1.81	2.00362
Hif	33–50	2.84	2.00358
Hef1	50–70	4.59	2.00359
Hef2	70–90	4.03	2.00356
Hef3	90–110	4.13	2.00360
Hif1	110–130	6.41	2.00355
Hif3	150–170	4.80	2.00355
Hif5	190–210	4.00	2.00359
Hif7	230–250	4.17	2.00353
Hif8	250–265	5.61	2.00356
North tundra			
Fibric Folic Cryic Histosol (P-4)			
O	0–2	2.52	2.00362
Hi1	2–10	2.90	2.00366
Hi2	10–20	3.05	2.00367
He1	20–30	3.83	2.00372
Hif1	30–40	3.31	2.00371
Hif3	50–60	5.28	2.00357
Hef2	80–100	5.04	2.00359
Hef4	120–133	7.48	2.00358
Hef5	133–150	3.78	2.00355

^1^ CPC—concentration of paramagnetic centres. ^2^—the absolute error for determination of the g-factor is 0.00020.

**Table 2:** Analysis of principal components for HA parameters.

Principal Components	Eigenvalues	% of Total Variance	Cumulative Eigenvalues	Cumulative % of Variance
PC1	8.80	46.34	8.80	46.34
PC2	3.21	16.87	12.01	63.21
PC3	1.55	8.14	13.56	71.35

**Table 3:** Properties of raw peats and HAs.

Horizon and Depth, cm	Peat Soils Characteristics			HA Characteristics													
	*R* ^1^, %	pH H_2_O	Eh ^2^, mV	C Atom Content of Molecular Fragments ^3^, %								Mole Fractions ^4^, %			*Mn* ^5^, kDa	Atomic Ratios	
				C,H-alkyl	O-CH_3_	O,N-alkyl	C,H-arom	O,N-arom	Carboxyl	Quinone	Carbonyl	*x*(HMF)	*x*(MMF)	*x*(LMF)		H/C	O/C
Forest tundra																	
Hemic Folic Cryic Histosol (P-1)																	
Hi 0–10	22.5	3.75	351	26.4	7.5	22.1	20.7	8.3	11.2	1.1	2.6	3.7	28.2	68.1	21.8	1.16	0.54
He1 10–20	37.5	3.70	358	27.3	7.5	20.5	21.6	8.9	11.1	1.1	2.0	3.0	27.2	69.8	18.5	1.10	0.55
He2 20–40	35.0	3.74	357	27.7	7.5	21.5	21.0	8.5	11.4	1.0	1.4	1.8	23.1	75.1	12.2	1.07	0.53
Hef1 40–60	32.5	4.34	304	23.9	7.4	19.8	23.4	9.8	11.4	1.3	3.1	1.2	22.8	76.0	9.3	1.06	0.56
Hef2 60–80	32.5	4.86	268	22.2	7.4	19.5	25.2	11.4	11.5	0.6	2.1	0.8	21.4	77.8	7.6	0.98	0.58
Hef3 80–100	37.5	4.94	248	19.7	8.2	19.7	26.1	12.0	10.4	1.3	2.5	1.0	19.2	79.9	7.4	0.92	0.41
Hef4 100–150	35.0	4.95	246	23.0	9.2	18.3	24.6	11.0	10.7	1.0	2.2	1.4	20.0	79.0	9.9	0.90	0.38
Hef5 150–175	37.5	5.37	273	22.4	8.2	17.7	25.4	11.2	10.9	1.7	2.4	1.7	28.6	69.7	12.6	0.94	0.48
Chfg 175–200	50.0	5.33	259	24.9	8.2	16.7	25.1	10.0	11.3	1.0	2.8	2.3	32.9	64.8	16.1	0.98	0.49
South tundra																	
Fibric Folic Cryic Histosol (P-2)																	
O 0–10	3.8	3.89	259	35.0	7.9	27.1	13.6	6.2	8.4	0.7	1.1	12.2	34.0	53.8	53.7	1.21	0.44
Hi1 10–15	10.0	3.62	275	33.3	7.9	26.5	15.0	6.6	8.7	0.8	1.2	8.6	33.1	58.3	37.9	1.21	0.44
Hi2 15–20	10.0	3.70	260	29.8	8.3	29.7	14.7	5.9	9.7	0.7	1.2	3.8	25.3	70.9	18.3	1.16	0.48
Hi3 20–26	10.0	3.71	275	24.5	7.3	32.7	15.4	6.3	10.7	1.2	1.9	3.8	27.0	69.1	18.8	1.03	0.52
Hif1 26–40	25.0	3.85	286	18.9	7.5	23.2	25.5	10.5	10.9	1.1	2.4	3.2	29.5	67.3	16.7	0.78	0.48
Hif2 40–50	27.5	4.15	270	18.5	7.9	23.6	25.4	10.6	10.7	1.1	2.2	1.8	17.9	80.3	9.6	0.87	0.47
Hif3 50–60	27.5	4.60	234	19.0	8.4	22.8	26.5	11.2	9.5	0.9	1.7	1.1	15.4	83.5	6.9	0.90	0.44
Hef1 80–100	35.0	5.19	200	16.9	8.7	22.0	28.4	11.9	9.5	0.9	1.7	0.6	13.1	86.3	4.9	0.92	0.43
Hef2 120–140	25.0	5.46	173	19.8	9.0	20.7	28.2	12.0	8.0	1.0	1.4	0.7	13.5	85.9	5.1	0.95	0.41
Hef3 160–180	22.5	5.30	186	20.0	9.4	21.4	27.0	11.7	8.4	0.8	1.4	1.4	17.4	81.2	8.2	1.01	0.42
Hif4 200–220	25.0	5.12	177	15.9	9.2	21.2	30.4	13.3	7.9	0.8	1.3	0.7	11.5	87.8	4.9	0.95	0.42
Hif5 240–260	50.0	5.19	163	22.0	9.5	20.2	27.1	10.3	9.6	0.6	0.8	0.9	23.7	80.8	7.6	0.95	0.46
Hif6 260–280	50.0	4.57	193	21.6	9.6	20.9	27.1	10.3	8.9	0.5	1.1	1.2	22.2	76.7	8.2	0.94	0.45
Ecoton north tundra—south tundra																	
Hemic Folic Cryic Histosol (P-3)																	
Hi 0–10	20.0	3.72	316.5	31.3	7.8	26.9	15.6	6.7	9.8	0.6	1.3	6.2	30.5	63.4	29.1	1.19	0.46
He 10–20	35.0	3.84	317	34.3	7.7	20.1	18.6	6.8	10.3	0.7	1.5	2.9	27.9	69.2	15.6	1.04	0.45
Hi1 20–30	22.5	3.84	285	35.5	8.0	16.4	20.5	7.4	10.3	0.6	1.3	2.4	27.6	69.9	14.1	0.99	0.44
Hi2 30–33	20.0	3.94	293	22.7	8.7	23.4	22.7	9.7	10.3	0.8	1.7	4.0	29.0	67.1	19.7	0.88	0.48
Hif 33–50	25.0	4.60	226	24.4	7.7	22.4	23.3	9.6	10.3	0.6	1.7	3.6	28.3	68.1	20.7	0.89	0.48
Hef1 50–70	40.0	5.23	188	21.3	8.1	22.0	25.2	10.1	10.6	0.9	1.9	3.4	27.6	69.0	19.7	0.89	0.47
Hef2 70–90	40.0	5.46	166	21.7	8.2	21.6	25.4	10.4	10.4	0.6	1.7	1.9	20.0	78.1	10.6	0.89	0.46
Hef3 90–110	40.0	5.50	160	24.6	8.8	22.3	23.4	8.9	10.2	0.7	1.2	3.7	29.8	66.5	18.7	0.93	0.47
Hif1 110–130	25.0	5.61	161	20.1	9.6	20.8	28.1	12.1	8.0	0.4	0.9	1.4	16.3	82.3	8.0	0.90	0.42
Hif3 150–170	20.0	5.52	196	19.6	10.2	21.4	28.7	12.2	7.0	0.2	0.6	1.2	16.8	82.0	7.2	0.94	0.40
Hif5 190–210	20.0	5.51	188	18.2	10.3	21.9	29.3	12.9	6.6	0.3	0.6	1.1	16.6	82.4	6.7	0.93	0.40
Hif7 230–250	25.0	5.73	185	18.0	10.1	20.6	28.8	12.7	7.9	0.7	1.1	1.2	17.6	81.2	7.5	0.96	0.37
Hif8 250–265	27.5	5.31	193	21.3	8.8	20.3	27.7	11.4	8.9	0.6	1.1	0.9	13.8	85.3	5.8	0.87	0.43
North tundra																	
Fibric Folic Cryic Histosol (P-4)																	
O 0–2	5.0	4.18	233	34.7	3.5	30.9	1.9	8.9	7.8	0.0	12.4	9.3	23.0	67.7	39.2	1.09	0.46
Hi1 2–10	15.0	4.20	233	28.9	6.8	29.2	15.8	7.0	9.8	0.9	1.6	5.7	27.7	66.6	25.7	1.10	0.47
Hi2 10–20	17.5	4.21	251	31.1	6.7	24.5	18.0	7.1	9.7	0.9	1.9	3.9	29.9	66.3	19.5	1.01	0.45
He1 20–30	37.5	4.24	260	30.6	6.8	23.4	18.5	7.1	10.2	1.1	2.3	3.5	30.2	66.3	17.9	1.06	0.44
Hif1 30–40	15.0	3.88	247	30.8	6.9	20.6	19.9	8.1	10.2	1.2	2.3	3.4	33.5	63.1	17.7	1.03	0.42
Hif3 50–60	17.5	3.92	232	36.2	7.4	19.9	17.5	6.6	9.6	1.1	1.6	4.1	31.0	67.1	20.1	1.09	0.42
Hef2 80–100	37.5	4.41	175	23.2	7.5	20.0	22.7	9.6	12.3	1.6	3.0	1.8	23.3	74.9	10.6	0.97	0.46
Hef4 120–133	42.5	5.37	121	21.6	7.5	18.3	25.8	11.6	11.5	1.4	2.4	1.4	20.2	78.4	8.8	0.88	0.46
Hef5 133–150	45.0	5.58	135	27.5	7.0	17.1	23.5	9.3	11.5	1.4	2.8	1.8	35.5	75.7	12.8	0.92	0.47

^1^ *R*—degree of peat decomposition. ^2^ Eh—redox potential. ^3^ CP/MAS ^13^C NMR spectroscopy data. ^4^ HMF, MMF and LMF—high, medium and low molecular fractions. ^5^ *Mn*—number average molecular weights.

## Data Availability

The data of molecular weight distributions of HAs have been obtained from the “Institute of Biology, Komi Science Center”.
